# Exposure to Corticosterone Affects Host Resistance, but Not Tolerance, to an Emerging Fungal Pathogen

**DOI:** 10.1371/journal.pone.0163736

**Published:** 2016-09-30

**Authors:** Julie Murone, Joseph A. DeMarchi, Matthew D. Venesky

**Affiliations:** Department of Biology, Allegheny College, Meadville, Pennsylvania, United States of America; Imperial College Faculty of Medicine, UNITED KINGDOM

## Abstract

Host responses to pathogens include defenses that reduce infection burden (i.e., resistance) and traits that reduce the fitness consequences of an infection (i.e., tolerance). Resistance and tolerance are affected by an organism's physiological status. Corticosterone (“CORT”) is a hormone that is associated with the regulation of many physiological processes, including metabolism and reproduction. Because of its role in the stress response, CORT is also considered the primary vertebrate stress hormone. When secreted at high levels, CORT is generally thought to be immunosuppressive. Despite the known association between stress and disease resistance in domesticated organisms, it is unclear whether these associations are ecologically and evolutionary relevant in wildlife species. We conducted a 3x3 fully crossed experiment in which we exposed American toads (*Anaxyrus* [*Bufo*] *americanus*) to one of three levels of exogenous CORT (no CORT, low CORT, or high CORT) and then to either low or high doses of the pathogenic chytrid fungus *Batrachochytrium dendrobatidis* (“*Bd*”) or a sham exposure treatment. We assessed *Bd* infection levels and tested how CORT and *Bd* affected toad resistance, tolerance, and mortality. Exposure to the high CORT treatment significantly elevated CORT release in toads; however, there was no difference between toads given no CORT or low CORT. Exposure to CORT and *Bd* each increased toad mortality, but they did not interact to affect mortality. Toads that were exposed to CORT had higher *Bd* resistance than toads exposed to ethanol controls/low CORT, a pattern opposite that of most studies on domesticated animals. Exposure to CORT did not affect toad tolerance to *Bd*. Collectively, these results show that physiological stressors can alter a host’s response to a pathogen, but that the outcome might not be straightforward. Future studies that inhibit CORT secretion are needed to better our understanding of the relationship between stress physiology and disease resistance and tolerance in wild vertebrates.

## Introduction

Hosts responses to pathogens can be broken into two strategies: those that prevent pathogen colonization or reduce infection burden (i.e., resistance) and those that reduce the fitness consequences of an infection (i.e., [1, 2]). Resistance reduces host infection burden and can thereby reduce disease prevalence within populations of hosts. In doing so, host resistance imposes negative selection pressures on pathogens, which can potentially increase their virulence within a population [3]. In contrast, tolerance responses mitigate the fitness consequences association with an infection (e.g., reducing self-damage from immune responses). Tolerance is measured as the slope of the relationship between the change in fitness and change in parasite burden [1, 2]. The fitness of tolerant host genotypes are relatively unaffected by an increasing pathogen burden [2]. Thus, in contrast to resistance responses, tolerance responses do not reduce or eliminate pathogen infection burdens and thus are hypothesized to increase pathogen prevalence within a host population while having neutral or positive selection pressures on pathogen virulence (but see [3, 4]). Although host genotypes vary naturally in their resistance and tolerance to infection, these responses are often also mediated by environmental conditions in which hosts and pathogens interact (reviewed in [5]). In addition, host traits such as sex [6], age/developmental stage [7], nutritional condition [8], and physiological state [9] are known to affect host immunocompetence and thus their resistance and tolerance to pathogens.

These context dependent effects on host immunity, coupled with a growing interest in differentiating between host resistance and tolerance in animal-pathogen systems, has generated considerable interest in the relationship between host condition and disease. Stress hormones, such as glucocorticoids, are associated with host condition because they are released during periods of stress and can have profound effects on the physiological state of vertebrates. For instance, corticosterone (hereafter “CORT”; the primary amphibian stress hormone) mobilizes energy reserves, which are often needed during the stress response [10]. In addition, CORT can alter the strength, distribution, and character of host immune defenses [11] and can mediate their responses to pathogens [12].

When CORT is chronically elevated, it typically reduces host resistance to infection [12] by limiting immunological defenses (glucocorticoids can also enhance immune functions in certain contexts [13]). For instance, inflammatory responses promote host resistance because they prevent the establishment and persistence of pathogens, even those for which a host does not have any immune memory. Inflammation, however, is dampened when CORT remains chronically high [11] and can thus reduce host resistance. Some evidence suggests that CORT can promote anti-inflammatory cytokines that reduce host damage associated with inflammation [14], which is thought to promote tolerance to pathogens [15]. Because innate immune responses begin with inflammatory responses and inflammation is an effective response to a diversity of pathogens, CORT is particularly relevant to ecological and evolutionary research questions targeted at host-pathogen interactions.

Despite knowing the effects of CORT on physiological and immunological responses of domesticated animals, few studies have manipulated this stress hormone to test how CORT mediates host-pathogen interactions using wild vertebrates (but see [16, 17–19]). Here, we present the results of a laboratory experiment in which we examined how CORT affects amphibians’ resistance and tolerance to *Batrachochytrium dendrobatidis* (*Bd*). *Bd* is a fungal disease of amphibians that causes the disease chytridiomycosis. Amphibians that develop chytridiomycosis generally undergo hyperkeratosis (the thickening of the *stratum corneum*), which disrupts cutaneous function and can lead to cardiac arrest [20]. *Bd* has been implicated in the declines of hundreds of amphibian species worldwide [21]. *Bd* generally causes high mortality in many species of amphibians but some individuals and species are known to be resistant or tolerant to infection [22–24]. In addition, amphibian resistance to *Bd* can be dependent on host condition [25, 26] and there is an association between *Bd* infection CORT release in amphibians [27–30], suggesting that host physiological condition might mediate resistance or tolerance to *Bd*.

We utilized a 3 x 3 fully factorial laboratory experiment in which we exposed American toads (*Anaxyrus* [*Bufo*] *americanus*) to one of three doses of CORT (high, low, and none) and then challenged them with *Bd* (high, low, and none) and measured how changes in their survival, resistance, and tolerance. We predicted that toads exposed to high doses of *Bd* would have higher *Bd* infections and mortality compared to toads given low doses (i.e., decreased resistance). We did not hypothesize that *Bd* dose would interact with CORT dose and thus we predicted and that the effects of *Bd* dose would occur independent of their exposure to CORT. If CORT is immunosuppressive the doses we used in our experiment, we predicted that exposure to CORT would reduce toad resistance to *Bd* (measured by infection abundance) and that *Bd*-induced mortality would increase as a function of increasing CORT dose. Because CORT is thought to promote tolerance to pathogens, we predicted that toads in the high CORT treatment would have higher tolerance to *Bd* than toads given low or no CORT (i.e., the slope of the relationship between change in fitness and *Bd* abundance would be negative for toads in the low or no CORT but flat for toads in the high CORT treatment).

## Methods

### Animal collection and husbandry

We collected recently hatched American toad (*Anaxyrus* [*Bufo*] *americanus*) tadpoles (stage 26–27 [31]) from a pond in Crawford County, Pennsylvania. Tadpoles were transported to the laboratory and immediately separated into five plastic containers (35.6 x 20.3 x 11.7 cm) at a density of approximately 6 tadpoles/L. The containers were gently aerated and were held in an environmental chamber at 28°C (± 1°C) on a 12: 12 light: dark photoperiod. The tadpoles were fed an *ad libitum* diet that consisted of a mixture of Sera Micron® (an algal based food) and spinach 4–5 times per week. We changed approximately 75% of the water from each container on a weekly basis.

The tadpoles completed metamorphosis between 02 June 2014 and 09 June 2014. Throughout the larval stage, we checked the containers 3 x each day and placed individuals with 4 legs into vented rectangular plastic containers (12.5 x 10 x 5 cm) that contained a tri-fold paper towel and approximately 15 mL of aged tap water. The containers were placed at a 20° angle so that the metamorphosing toad had access to water and also a damp substrate. Once the toad had fully absorbed its tail, we considered the individual a “juvenile” and moved it to an environmental chamber at 18°C (±1°C) on a 12: 12 light: dark photoperiod. Juvenile toads were housed in vented circular plastic containers (7.5 cm deep and 11 cm diameter) on tri-fold paper towels that were soaked with 12mL of aged tap water. Juvenile toads were fed fruit files (*Drosophila* sp.) *ad libitum* 2–3 times per week until they were large enough to consume crickets. At that point, and throughout the remainder of the experiment, they were fed 4–6 crickets (0.318–0.635 cm) per week. We replaced the paper towel bedding with fresh bedding on a weekly basis.

### Experimental design

Our experiment utilized a 3 x 3 fully factorial design in which we randomly assigned 90 juvenile toads to one of the 9 resulting treatments (n = 10/treatment) and tested for main and interactive effects of CORT and *Bd* on toad survival, resistance, and tolerance. On 23 June 2014, we exposed toads to one of three CORT treatments: 0.1 μM solution, 0.01 μM solution, or an ethanol control. We followed the protocol of Searle et al. [28] to make our CORT solutions. In brief, we dissolved 0.0056 g or 0.0561 g of CORT into one of two vials that contained 9.72 mL of ethanol (representing our low and high CORT treatments). We then applied the designated CORT treatment (or ethanol) and 15 mL of water to a fresh tri-fold paper towel every other day for 10 consecutive days (i.e., 5 applications). This volume was sufficient to fully saturate the paper towel so that the toad was always in contact with solution. The application of exogenous CORT directly on the bedding of post-metamorphic anurans at similar levels and durations (0.5 μM daily for 15 days) has been shown to elevate circulating levels of whole-body CORT to physiologically relevant levels [28]. Twenty four hours after the last CORT application, we conducted a bedding change and placed each toad on a paper towel that contained 12 mL of aged tap water. The toad was held on this paper towel for an additional 24 hours to avoid measuring any remaining exogenous CORT from the bedding in our water-borne CORT assay (see below).

### Quantification of water-borne CORT

We extracted and quantified water-borne CORT following the protocol of Gabor et al. [32], which has been validated on a number of species of frogs, toads, and salamanders [27, 32, 33] and also in fish (reviewed in [34]). After the toads were in their focal containers for 25 hours, we transferred toads to individual, sterilized, Petri® dishes (diameter: 140 mm; depth: 25 mm) filled with 50 mL of water for 60 m. All toads were sampled at the same day and time to avoid their potentially confounding effects. The water samples were stored at -20°C until the extraction of the water-borne CORT.

We randomly selected 30 toads (n = 10 from each of the three CORT treatments) and analyzed the total (free + conjugated) CORT released in the water samples. We first thawed each water sample and extracted CORT from the entire sample by passing water through a sterilized 60mL syringe (309653; Becton Dickinson, NJ, USA) into C18 solid phase extraction columns (SEP-PAK, Waters Inc.). Prior to use, the columns were primed with 4 mL HPLC-grade methanol and 4 mL of millipore filtered water. We eluted the columns with 4 mL methanol into borosilicate vials and the methanol was evaporated under a stream of nitrogen gas at 37°C using an Evap-O-Rac (Cole-Palmer). Samples were re-suspended in 20 μL of 100% ethanol, vortexed for 1 m, and then in 380 μL EIA buffer (Cayman Chemicals Inc., MI, USA) for a final volume of 400 μL. CORT was measured using an enzyme-immunoassay (EIA) kit (Cayman Chemicals Inc.). All samples were run in duplicate. All CORT data are presented as pg/sample (pg/mL multiplied by 0.40 mL, which accounts for the amount of EIA buffer used to reconstitute the sample).

### *Bd* exposures

The *Bd* isolate that we used in our experiment (JEL 660) was isolated from a wild-collected anuran in Ohio and cryopreserved at the University of Maine since isolation. The stock culture used in our experiment has been held in 1% tryptone broth at 5C in the laboratory at Allegheny College since 2014. Throughout this time, we transferred the culture to new media every 4–5 months (approximately 3 times prior to the start of the experiment).

Immediately after removing the toads from their Petri® dishes (above), we placed them back into their respective focal container and exposed them to one of three *Bd* treatments by pipetting 2.0 mL of tryptone broth that contained either 8.0x10^4^ zoospores, 2.0x10^4^ zoospores, or a sham exposure that did not contain any zoospores onto the dorsal side of each toad. Excess innocula was allowed to drip onto the paper towel substrate, which formed a thin layer of innocula. The toads were held on this substrate for 48 hours, after which we replaced the *Bd*-exposed (or tryptone) bedding with fresh bedding as described above. To prevent accidental transfer of *Bd* zoospores between toads, the experimenter used a different nitrile glove for each animal when conducting the bedding changes. Throughout the experiment, all equipment was either autoclaved or placed in a 10% bleach solution to kill *Bd* [35].

*Bd* is implicated in global amphibian declines and infections are expected to induce symptoms of chytridiomycosis and also death. Throughout the duration of the experiment, we monitored the toads twice daily for a proper righting response or for mortality. Animals that did not exhibit a righting response were deemed pre-moribund and euthanatized to alleviate pain and discomfort. Toads that lost their righting response were placed individually in a glass beaker that contained approximately 50 mL of an overdose of MS222 (≥ 250 mg/L) to anesthetize and euthanize. Any dead toad or toad that lost its righting response was massed (to the nearest 0.01 g), immediately swabbed for *Bd* quantification (see below), and placed in a -20°C freezer. We terminated the experiment 28 days after the toads were exposed to *Bd*, at which all surviving toads were massed and then euthanatized with an overdose of MS222 (described above).

### Swabbing and qPCR details

*Bd* infection on the toads was measured by swabbing each animal on Day 7 post-exposure or on the day a toad died. We passed a sterile swab (Medical Wire & Equipment; MW 113) across the dorsal surface of each toad a total of 10 times. We also recorded the mass of each toad (to the nearest 0.01 g) on Day 7. To prevent cross-contamination with *Bd* or *Bd* DNA, the experimenter used a different pair of nitrile gloves while swabbing and massing each toad.

Our DNA extractions and qPCR analyses followed the methods of Boyle et al. [36] and modified by Hyatt et al. [37]. Test samples were run singly instead of triplicate to control costs, as recommended by Kriger et al. [38]. We added TaqMan® Exogenous Internal Positive Control (Exo IPC) Reagents (Applied Biosystems, Foster City, CA) to every reaction well to assess inhibition of the PCR reaction [37]. The Exo IPC system uses a standardized concentration of an artificial DNA sequence that is added to each reaction well with its own set of primers and a separate fluorescent probe. The strength of this reaction is used to assess overall reaction inhibition. Extractions were diluted 1:100 and processed in an Applied Biosystems Step One Real-time PCR system. None of our samples were inhibited and thus did not require any further dilution. We considered infection intensity as the number of *Bd* zoospore equivalents per sample. Zoospore equivalents were calculated by multiplying the genome equivalent values generated by the qPCR assay by 80, which accounts for the fact that the DNA from the swabs was diluted 80-fold during extraction and qPCR preparation. We considered a sample *Bd* positive when zoospore equivalents were ≥ 1 [39, 40].

### Statistical analyses

**Fig 1:**
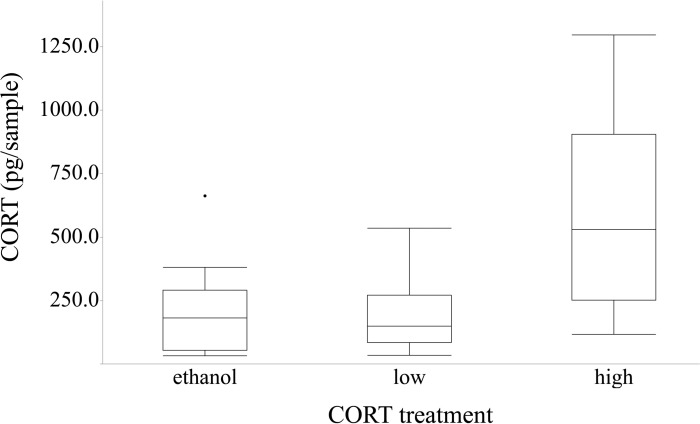
Corticosterone (CORT) release rates in toads. Corticosterone (CORT) release rates of American toads (*Anaxyrus* [= *Bufo*] *americanus*) exposed to either an ethanol control, low CORT (0.01 μM), or high CORT (0.1 μM). Toads in the high CORT treatment had significantly higher CORT than the toads in either the ethanol or low CORT treatments.

Our first statistical analyses tested for differences in CORT release in the water (pg/sample) in toads from the three CORT treatments (categorical responses: none, low, and high). We added mass (taken that day) as a co-variate in the analysis to account for size differences in the toads. We used a liner model ("lm" function in R statistical software) and assessed statistical significance (*p* < 0.05) using the "Anova" function in the "car" package of R statistical software. Although the toads given high CORT had higher CORT release into the water compared to the other two treatments, there was no difference between the CORT release in the water between the toads given no CORT and low CORT (see Results and Fig 1), we combined the toads from the no CORT and low CORT treatments into a single treatment in the following statistical analyses (see S1 Appendix for the statistical procedures and results using three CORT treatments instead of two).

We used the “coxph” function in the “survival” package in R, which uses a Cox proportional hazards regression model. This statistical model censors all individuals surviving on day 28 to account for our lack of information about their true times of death, a standard approach to down-weight the influence of these individuals in the survival analysis. In this analysis, we tested for main and interactive effects of the categorical response variables CORT (none/low and high) and *Bd* dose (none, low, and high) on toad survival. We assessed statistical significance (*P* < 0.05) using the “Anova” function in the “car” package of R.

Our next statistical models compared differences in host resistance using *Bd* abundance (i.e., the infection intensity of all *Bd*-exposed toads, including those with a "0" infection burden) as a response variable. Our first model tested for differences in resistance on Day 7. Here, we excluded the *Bd*-exposed toads that died prior to Day 7 (n = 11). We tested for the main and interactive effects of CORT exposure (none/low and high) and *Bd* dose (low and high) on our response variable (log transformed *Bd*+1). By definition, "resistance" refers to the ability of a host to prevent or eliminate an infection [2] and non-exposed animals are not considered in analyses of resistance because one would not expect them to have any pathogen burden. We included the pre-experiment mass of each toad and the number of days that the toad survived as covariates in the analysis because large toads could have more *Bd* simply because of their body size. We used number of days alive to avoid the potentially confounding factor of differences in the number of days that *Bd* could grow on the toads. In our second model, we used the maximum *Bd* infection, which we defined as the maximum *Bd* abundance we detected on a swab from either Day 7 or when the toad died (if it died). We used the same predictors as described above. We assessed statistical significance (*P* < 0.05) in both models using the "Anova" function in the "car" package of R statistical software.

To test for differences in tolerance, we used the percentage of mass change throughout the experiment (or when the toad died) as a proxy for host fitness because anuran metamorphic mass is generally a good predictor of fitness [41]. To account for differences in vigor (i.e., fitness in the absence of parasite exposure), we included all of the toads that were given a sham (control) *Bd* dose in our analysis. We used the "lm" function in R and tested for main and interactive effects of CORT exposure (none/low and high) and log-transformed maximum detected *Bd* abundance (i.e., the highest *Bd* infection value obtained from Day 7 or the date of death) on host fitness. We also included the number of days alive as a covariate in the analysis. We assessed statistical significance (*p* < 0.05) using the “Anova” function in the “car” package of R.

### Ethical approval

All applicable institutional and national guidelines for the care and use of animals were followed. The protocol was approved by the Institutional Animal Care and Use Committee (through the Animal Research Committee) of Allegheny College. American toad tadpoles were collected through a Scientific Collectors Permit granted to MDV through the PA Fish and Boat Commission.

## Results

The CORT release into the water differed among treatment groups (ANOVA, *F*_2,26_ = 7.505, *P* = 0.003), even after controlling for the effects of mass (*F*_1,26_ = 0.261, *P* = 0.614) on CORT release. Toads exposed to ethanol and a low dose of CORT had similar CORT release and toads exposed to a high dose of CORT had a higher CORT release than the other two treatments (Fig 1). The intra-assay coefficient of variation for the plate was 12.5% (determined by the dividing standard deviation for each duplicate sample by the sample mean to obtain a sample coefficient of variation and then averaging all of the individual coefficient of variation values) and the standards had a good fit to the standard curve (*r*^2^ = 0.9896).

None of the toads that were given a sham exposure were infected with *Bd* (0% prevalence and 0.00 zoospores). The average infection intensity of toads infected with *Bd* (excluding the exposed but non-infected toads) was 189.29 (SE ± 69.82) and 1089.11 (SE ± 367.05) zoospores in the low and high treatments, respectively.

**Fig 2:**
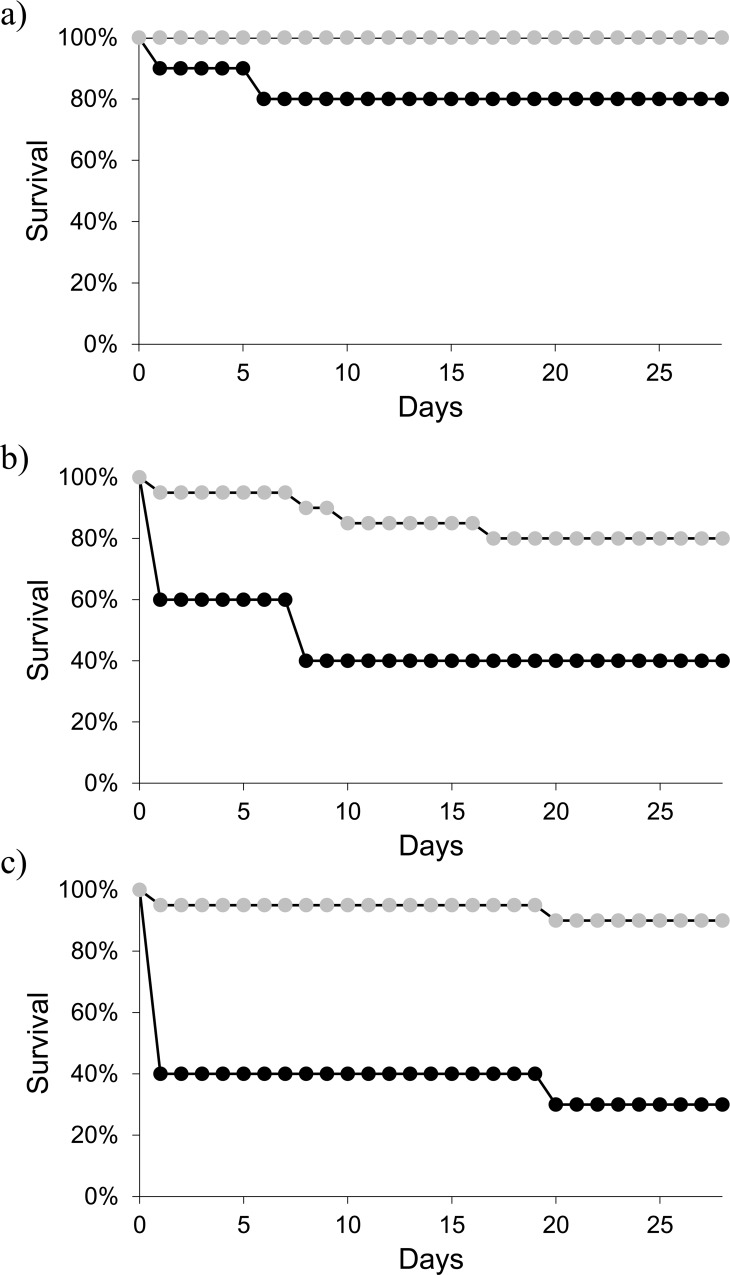
Survival plots of toads. Survival plots of American toads (*Anaxyrus* [= *Bufo*] *americanus*) exposed to (*a*) a control treatment without *Batrachochytrium dendrobatidis* ("*Bd*), (*b*) a low dose of *Bd*, and (*c*) a high dose of *Bd*. Grey circles represent toads exposed to the ethanol/low CORT dose (*n* = 20 per panel); black circles represent toads exposed to a high CORT dose (*n* = 10 per panel). Exposure to *Bd* and CORT independently reduced survival, but the two factors did not interact to affect survival.

Exposure to *Bd* increased toad mortality (Cox, *X*^2^ = 12.204, *P* = 0.002) and the overwhelming majority of the toads that died during the experiment died within 24 hours of exposure to *Bd* (Fig 2B and 2C). Irrespective of their CORT treatment, toads that were exposed to *Bd* had higher mortality (~33% in the low *Bd* treatment and ~37% in the high *Bd* treatment compared to non-exposed toads (~6%). Toads that were exposed to CORT had significantly higher mortality than did toads not exposed to CORT (Cox, *X*^2^ = 17.848, *P* < 0.001). Although the interaction between *Bd* dose and CORT was nonsignificant (Cox, *X*^2^ = 1.494, *P* = 0.474). See S1 Appendix and S1 Fig for the results using three CORT doses.

**Fig 3:**
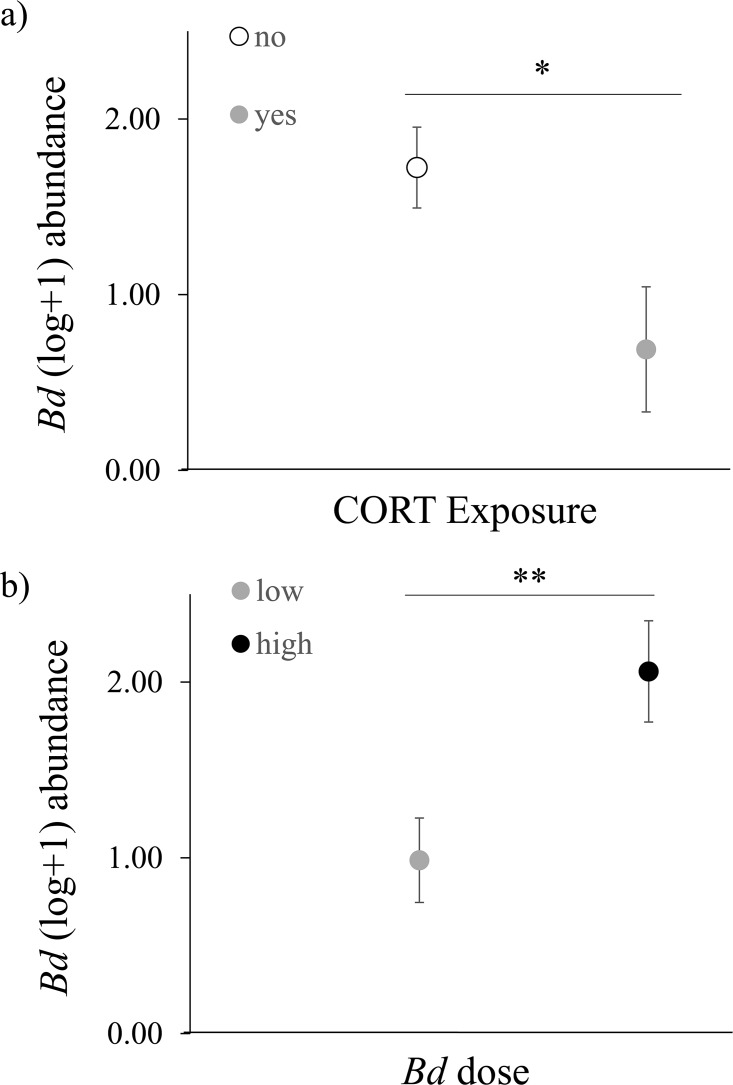
Effects of Corticosterone (CORT) on toad resistance. Significant main effects of (*a*) CORT exposure and (*b*) *Bd* dose on American toad (*Anaxyrus* [= *Bufo*] *americanus*) resistance, as measured by *Batrachochytrium dendrobatidis* (*Bd*) abundance. Values represent the average log-transformed *Bd* abundance for each treatment. Error bars indicate 1 S.E. Statistical differences between treatments are indicated as * = 0.026 and ** = 0.004. Sample sizes for the CORT exposure treatments were 38 and 10 (no CORT and CORT exposed, respectively). Sample sizes for the *Bd* dose treatments were 25 and 23 (low and high *Bd*, respectively).

When examining toad resistance to *Bd*, we detected quantitatively similar main and interactive effects of *Bd* dose and CORT when using the Day 7 swabs and the maximum *Bd* detected as response variables (Table 1 and S1 Table), indicating that our findings are robust. As such, we presented the results and figures using Day 7 data. *Bd* dose significantly affected toad resistance to *Bd* (Table 1) in a dose dependent fashion, where toads exposed to a high level of *Bd* had significantly higher *Bd* abundance compared to toads exposed to a low level of *Bd* (Fig 3A). We also found a significant main effect of CORT exposure on resistance to *Bd* (Table 1). Toads exposed to CORT had a lower *Bd* abundance compared to toads exposed to an ethanol control/low CORT (Fig 3B). Exposure to CORT and *Bd* dose did not significantly interact to affect resistance (Table 1). See S2 Fig for the results using three CORT doses.

**Table 1:** The main and interactive effects of CORT exposure (none/low, and high) and *Bd* dose (low, high) on toad resistance, as measured by Day 7 *Bd* abundance.

Predictor	*F* value	d.f.	*P* value
CORT exposure	4.974	1	0.026
*Bd* dose	8.443	1	0.004
Mass	1.578	1	0.194
Days Alive	1.044	1	0.213
CORT x *Bd*	1.172	1	0.296
Error		42	

**Fig 4:**
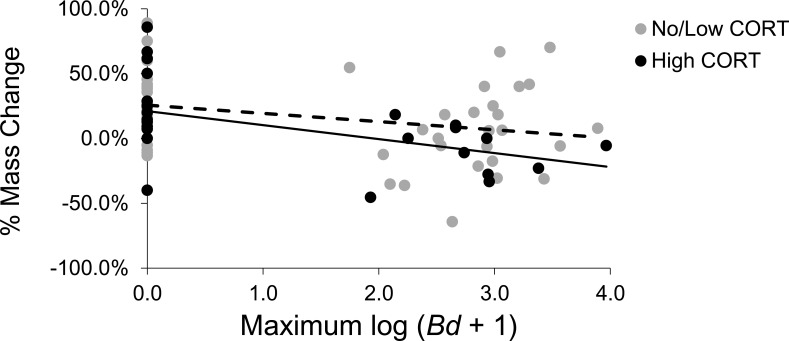
Effects of Corticosterone (CORT) on toad tolerance. The effect of CORT exposure on American toads (*Anaxyrus* [= *Bufo*] *americanus*) tolerance to *Batrachochytrium dendrobatidis* (*Bd*), as measured by the relationship between maximum *Bd* infection intensity and percentage of mass change.

There was a significant negative relationship between maximum *Bd* abundance and the percentage of mass change by toads (linear model, *F*_1,85_ = 6.183, *P* = 0.015), indicating that toads are not tolerant to *Bd*. However, exposure to CORT did not affect toad tolerance to *Bd* (linear model, CORT x max *Bd*: *F*_1,85_ = 0.176, *P* = 0.676; Fig 4). See S3 Fig for the results using three CORT doses.

All data are available in S1 Dataset.

## Discussion

The idea that glucocorticoids, such as CORT, are immunosuppressive when applied at high concentrations for long periods of time is one of central paradigms in disease ecology and eco-physiological. Although connections between stress, physiology, and immunology are well supported [11, 12], these principles have rarely been used to explain causal relationships between stress and disease outcomes in wild vertebrates. Our fully factorial laboratory experiment using field-collected American toads (*Anaxyrus* [*Bufo*] *americanus*) exposed to physiologically relevant doses of CORT and the pathogenic chytrid fungus revealed that CORT can mediate an outcome of infectious diseases.

Contrary to our predictions, and much of the literature on stress physiology disease risk, toads exposed to CORT had a lower *Bd* abundance compared to toads given no CORT/low CORT (Fig 3B). Although the association between glucocorticoids and their physiological consequences are frequently thought of as suppressive, glucocorticoids have many actions including permissive and preparative capacities [10]. The most plausible explanation for our finding was that our high CORT dose (0.1 μM) did not chronically elevate circulating levels of CORT in the toads, but were instead more similar to an acute stressor and were thus preparative. Preparative actions are thought to modulate an organism’s stress response by enhancing responsiveness to a subsequent stressor, such as infectious diseases [42]. Evidence from the mammal literature demonstrates that glucocorticoids can enhance the production of immunoglobulins [43], T cell function [44], and cytokine-mediated immune responses [45]. This rationale is also supported by a recent study by Tatiersky et al. [46] who found that injection of Northern leopard frogs (*Lithobates pipiens*) with CORT increased gene expression of cutaneous antimicrobial peptides, which are known to increase amphibian resistance to *Bd* [47–50]. Recent research in the amphibian-*Bd* model system also demonstrates that anuran resistance to *Bd* is also associated with leukocyte abundance and proliferation [22]. Circulating leukocytes can change in response to exogenous CORT [51] and thus changes in leukocyte abundance are another possible mechanism underlying our findings. Although we did not measure changes in any immune parameters in our experiment, the evidence outlined above provides possible CORT-mediated mechanisms for our finding. Future research that further test how glucocorticoids enhance (or suppress) specific immune responses in wild vertebrates are needed to fully understand the outcomes of disease.

Our result that *Bd* resistance is influenced by CORT is opposite the results presented in a similar paper in which Searle et al. [28] found that exposure to CORT did not affect *Bd* infections in three species of amphibians (including a toad of the same genus as the ones we used). However, there are a number of reasons for our different results. First, disease outcomes are context dependent [52] and our experiment used a different *Bd* isolate than did Searle et al. [28]. Each *Bd* isolate was regional to the location in which the respective host species were collected and likely represent ecologically and evolutionarily relevant host-pathogen interactions. *Bd* isolates differ in their virulence [53] and whether/how CORT mediates host resistance to *Bd* isolates with different virulence is unknown. Second, the majority of amphibians used by Searle et al. [28] were tadpoles whereas our study used metamorphic amphibians. Amphibian resistance to pathogens changes through development [7, 54], most likely due to the reorganization of the amphibian immune system after metamorphosis [55], which is mediated by glucocorticoids [56]. Thus, the different patterns observed across studies might be associated with different life stages. However, Searle et al. [28] did use metamorphic anurans in one experiment and also found no effect of CORT on *Bd* infection. This difference is most likely explained by the fact that our CORT doses differed (our dose of 0.1 μM versus 0.5 μM in 28). Lastly, our average infection intensity was at least three orders of magnitude higher than the juvenile anurans used in the former study (the average *Bd* infection intensity of the toads in our experiment was 1333.99 genome equivalents compared to 0.84 genome equivalents reported in [28]). One possible reason that Searle et al. [28] failed to detect a significant effect of CORT on host resistance was because of the lack of variation across the samples due to the fact that the frogs were infected with less than a single zoospore.

Tolerance is an important, yet often overlooked, aspect of an animal’s defense strategy. One way in which a host can increase their tolerance to disease is by reducing self-damage associated with inflammatory responses directed at pathogens (e.g., increasing anti-inflammatory cytokines or transforming growth factor beta which induce anti-inflammatory T-cells [15]). Although few studies have actually identified mechanisms of host tolerance in wild vertebrates, recent evidence links glucocorticoid-mediated anti-inflammatory responses with reductions in immunopathology as drivers of tolerance [57]. We found no evidence that CORT improved toad tolerance to *Bd*. In fact, if anything, the qualitative observation that exposure to CORT decreased toad tolerance to *Bd* (Fig 4) is opposite the predictions based on the literature. There is evidence that some species of amphibians respond to *Bd* by increasing inflammatory cells in the skin [58, 59] and genomic analyses have found that genes associated with inflammation are upregulated in *Bd*-infected amphibians [60, 61]. For instance, histological evidence of anurans infected with *Bd* show mild to moderate infiltrates of inflammatory cells within the dermis, occasionally extending into the epidermis [62]. Thus, although it is plausible that CORT-mediated anti-inflammatory responses could promote tolerance to *Bd*, our results do not support that hypothesis. More research is needed to understand the mechanisms of amphibian tolerance to *Bd*, including understanding the link between CORT, anti-inflammatory cytokines, and host tolerance.

Although infection with *Bd* significantly increased toad mortality compared to non-exposed toads, we did not find any differences in mortality between the low- and high-exposure treatments (Fig 2B and 2C) despite significant differences in average *Bd* abundance between these two treatments (Fig 3B). This result is even more surprising considering that previous researchers found dose-dependent patterns of mortality observed in recently metamorphosed toads of the same genus [63]. The *Bd*-infected toads that died in our experiment had an average infection intensity of 1470 zoospores (±530 SE), which is orders of magnitude lower than the 10,000 zoospore mortality threshold proposed by other researchers [64], which suggests that amphibian species or life-stage and *Bd* isolate, or their interaction, can cause result in amphibian mortality at much lower infection intensities.

In addition to the main effect of *Bd* on toad mortality, we found that toads exposed to exogenous CORT had higher mortality than toads exposed to no CORT/low CORT, irrespective of the *Bd* treatment. It is difficult to reconcile the notion that exposure to CORT can have beneficial (increased resistance to *Bd*; Fig 3) and negative (increased mortality; Fig 2) effects when applied at the same dose for the same duration of time. However, the patterns of mortality in the CORT treatments seem to be driven primarily by *Bd*. Of the 20 toads in the CORT treatment that were exposed to *Bd*, 13 died (n = 7 and 7 in the low and high *Bd* treatments, respectively; Fig 2B and 2C) whereas only 2 of the 10 toads that were not exposed to *Bd* but received a high dose of CORT died (Fig 2A). The fact that we did not detect a significant interaction between CORT and *Bd* on survival is likely an issue of statistical power. An alternative explanation to this pattern that is independent of *Bd* is that toads exposed to CORT experienced an increased metabolic rate [65, 66] and died from an energetic/caloric deficit. Our test subjects were recently metamorphosed amphibians and likely did not have a surplus of energetic reserves. Given this logic, one could predict that the toads exposed to CORT but died would experience a mass loss whereas toads that were exposed to high CORT but survived would gain mass. Although our sample size for these data is too limited to test statistically, our results follow this pattern: surviving toads that were exposed to CORT increased their mass by 21.6% compared to an 8.3% mass loss for toads that were exposed to CORT and died. Taken collectively with our data on CORT and *Bd* resistance, these findings could suggest that the toads healthy enough to survive exposure to CORT were also the ones that were the most resistant to *Bd*.

Understanding how physiological stressors affect host resistance and tolerance to pathogens is an essential component to the ecology of infectious diseases. However, few studies have tested how CORT affects host responses to pathogens using wild vertebrates (but see [16, 17–19]) and to our knowledge, our study is the first to extend this conceptual framework to tolerance to infection. Our results show that host physiological condition can change disease outcomes, but that these changes are not always straightforward. For example, exposure to high levels of CORT generally increased toad mortality, enhanced resistance to *Bd*, but did not affect tolerance. Our results emphasize the need for more studies that test how host physiological condition can affect resistance and tolerance to infection. In general, a larger sample size could help tease apart some of the patterns that we observed (e.g., an interaction between CORT dose and *Bd* on mortality, the effect of CORT on host tolerance, the effect of exogenous CORT exposure on CORT release) but lacked the proper statistical power to rigorously test. In addition, understanding how different exogenous doses of CORT relate to the various physiological levels of endogenous CORT will be important to understand whether hosts are in the reactive scope of their stress response. In addition, future studies that inhibit CORT secretion would help our understanding of the relationship between stress physiology and disease resistance and tolerance in wild vertebrates.

## Supporting Information

S1 Appendix Statistical analyses and results when using three CORT treatments. (PDF)

S1 Dataset Data used in our analyses. (CSV)

S1 Fig Survival plots of toads. Survival plots of American toads (*Anaxyrus* [= *Bufo*] *americanus*) exposed to (a) a control treatment without *Batrachochytrium dendrobatidis* (*Bd*), (b) a low dose of *Bd*, and (c) a high dose of *Bd*. Open circles represent toads exposed to the ethanol treatment (n = 10 per treatment), grey circles represent toads exposed to the low CORT treatment (n = 10 per treatment); black circles represent toads exposed to the high CORT treatment (n = 10 per treatment). Exposure to *Bd* and CORT independently reduced survival, but the two factors did not interact to affect survival.
(PDF)

S2 Fig Effects of Corticosterone (CORT) on toad resistance. Main effects of (a) CORT exposure and (b) *Batrachochytrium dendrobatidis* (*Bd*) dose on American toad (*Anaxyrus* [= *Bufo*] *americanus*) resistance, as measured by *Bd* abundance. Values represent the average log-transformed *Bd* abundance for each treatment. Error bars indicate 1 S.E.
(PDF)

S3 Fig Effects of Corticosterone (CORT) on toad tolerance. The effect of CORT exposure on American toads (*Anaxyrus* [= *Bufo*] *americanus*) tolerance to *Batrachochytrium dendrobatidis* (*Bd*), as measured by the relationship between maximum *Bd* infection intensity and percentage of mass change.
(PDF)

S1 Table The main and interactive effects of Corticosterone (CORT) exposure (none/low, and high) and *Batrachochytrium dendrobatidis* (*Bd*) dose (low, high) on toad resistance, as measured by maximum *Bd* abundance. (PDF)
