# Supplementary material for: Exposure to Corticosterone Affects Host Resistance, but Not Tolerance, to an Emerging Fungal Pathogen
Source: PLoS One. 2016 Sep 30;11(9):e0163736. doi: 10.1371/journal.pone.0163736 (PMC5045185; doi:10.1371/journal.pone.0163736)
Supplement: S1 Appendix — (PDF) [file pone.0163736.s001.pdf]

S1 Appendix. **Statistical Analyses and Results when Using Three CORT Treatments**

In the main text of the manuscript, we elected to condense the ethanol and low CORT treatments into a single categorical variable because there was no difference between the CORT release rates from American toads (Anaxyrus [= Bufo] americanus) in these two treatments. This appendix contains the description of our statistical procedures and the results from the analyses when we retained all three CORT treatment groups.

Statistical Analyses

In our first analysis, we used the “coxph” function in the “survival” package in R, which uses a Cox proportional hazards regression model. This statistical model censors all individuals surviving on day 28 to account for our lack of information about their true times of death, a standard approach to down-weight the influence of these individuals in the survival analysis. In this analysis, we tested for main and interactive effects of the categorical response variables CORT (none, low, and high) and *Bd* dose (none, low, and high) on toad survival. We assessed statistical significance ($p < 0.05$) using the “Anova” function in the “car” package of R.

Our next statistical models compared differences in host resistance using *Bd* abundance (i.e., the infection intensity of all *Bd*-exposed toads, including those with a "0" infection burden) as a response variable. Our first model tested for differences in resistance on Day 7. Here, we excluded the *Bd*-exposed toads died prior to Day 7 (n = 11). We tested for the main and interactive effects of CORT exposure (none, low and high) and *Bd* dose (low and high) on our response variable (log transformed *Bd*+1). We included the pre-experiment mass of each toad and the number of days that the toad survived as covariates in the analysis. We used number of

days alive to avoid the potentially confounding factor of differences in the number of days that *Bd* could grow on the toads. In our second model, we used the maximum *Bd* infection, which we defined as the maximum *Bd* abundance we detected on a swab from either Day 7 or when the toad died (if it died). We used the same predictors as described above. We assessed statistical significance ($p < 0.05$) in both models using the "Anova" function in the "car" package of R statistical software.

To test for differences in tolerance, we used the percentage of mass change throughout the experiment (or when the toad died) as a proxy for host fitness because anuran metamorphic mass is generally a good predictor of fitness. To account for differences in vigor (i.e., fitness in the absence of parasite exposure), we included all of the toads that were given a sham (control) *Bd* dose in our analysis. We used the "lm" function in R and tested for main and interactive effects of CORT exposure (none, low and high) and log-transformed maximum detected *Bd* abundance (i.e., the highest *Bd* infection value obtained from Day 7 or the date of death) on host fitness. We also included the number of days alive as a covariate in the analysis. We assessed statistical significance ($p < 0.05$) using the “Anova” function in the “car” package of R.

Results

Exposure to *Bd* increased toad mortality ($P = 0.002$) and the overwhelming majority of the toads that died during the experiment died within 24 hours of exposure to *Bd* (S1 Fig). Toads that were exposed to CORT had significantly higher mortality than did toads not exposed to CORT ($P < 0.001$). Although the interaction between *Bd* dose and CORT was nonsignificant ($P = 0.406$), it appears that the significant main effect of CORT exposure is being driven by mortality of toads that were exposed to CORT in the low and high *Bd* treatments.

When examining toad resistance to *Bd* as measured by *Bd* infection abundance on Day 7 (i.e., excluding the toads that died prior to Day 7), we found that the effect of *Bd* dose on toad resistance was significant (linear model, $F_{1,40} = 8.899$, $P = 0.004$; S2 Fig). We also found a marginally significant effect of CORT exposure on resistance to *Bd* (linear model, $F_{2,40} = 2.713$, $P = 0.078$; S2 Fig). Exposure to CORT and *Bd* dose did not significantly interact to affect resistance (linear model, $F_{2,40} = 1.609$, $P = 0.213$). Neither mass nor the number of days alive was a significant predictor of *Bd* resistance ($P > 0.150$ for each predictor). When examining toad resistance to *Bd* as measured by the maximum *Bd* abundance, we found that the effect of *Bd* dose on toad resistance was significant (linear model, $F_{1,52} = 10.061$, $P = 0.002$). We also found a significant effect of CORT exposure on resistance to *Bd* (linear model, $F_{2,40} = 4.234$, $P = 0.020$). Both of these effects occurred while statistically controlling for the significant effects of the covariates of mass and the number of days alive on *Bd* resistance (mass: $P = 0.047$; days alive: $P < 0.001$). Exposure to CORT and *Bd* dose did not significantly interact to affect resistance (linear model, $F_{2,40} = 0.751$, $P = 0.477$).

There was a significant negative relationship between maximum *Bd* abundance and the percentage of mass change by toads (linear model, $F_{1,83} = 5.950$, $P = 0.017$), indicating that toads are not tolerant to *Bd*. However, exposure to CORT did not affect toad tolerance to *Bd* (linear model, CORT x max *Bd*: $F_{2,83} = 1.170$, $P = 0.315$; S3 Fig).
