# Supplementary figures and images for: Exposure to Corticosterone Affects Host Resistance, but Not Tolerance, to an Emerging Fungal Pathogen
Source: PLoS One. 2016 Sep 30;11(9):e0163736. doi: 10.1371/journal.pone.0163736 (PMC5045185; doi:10.1371/journal.pone.0163736)

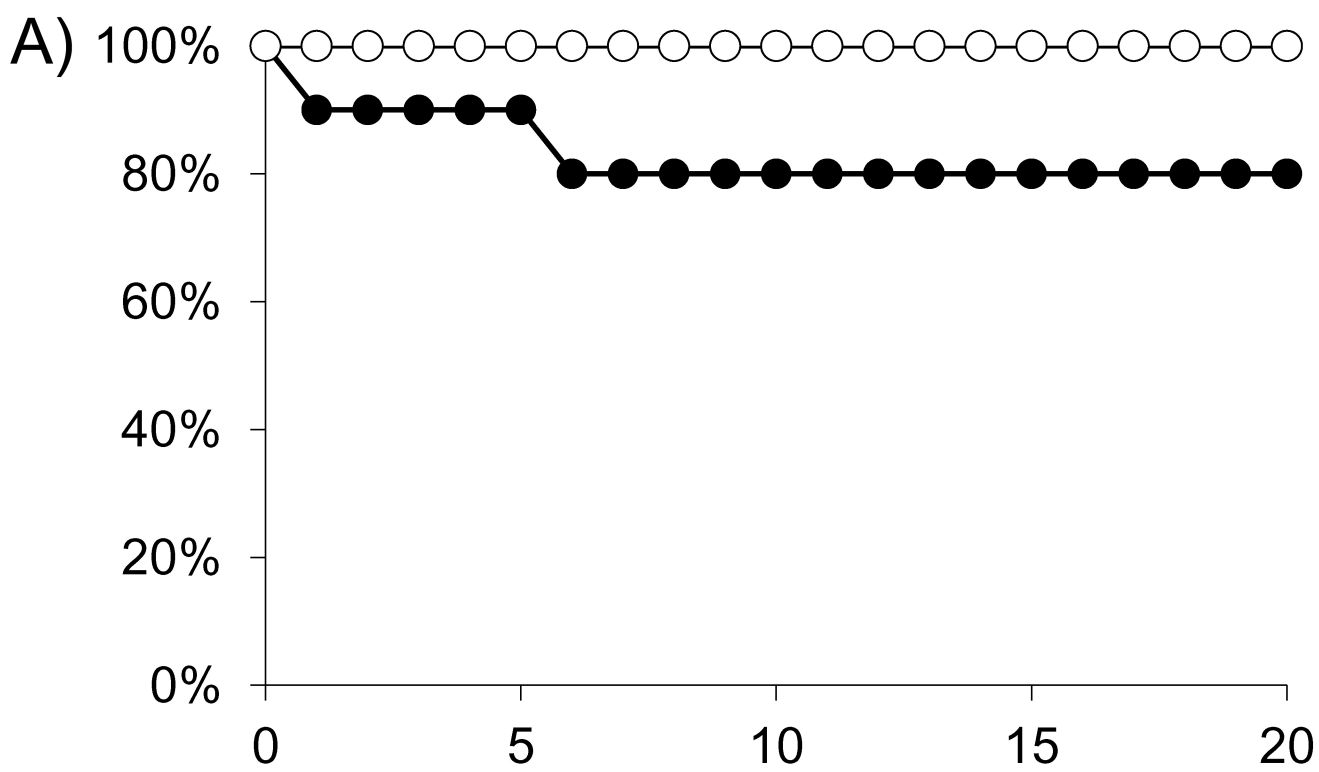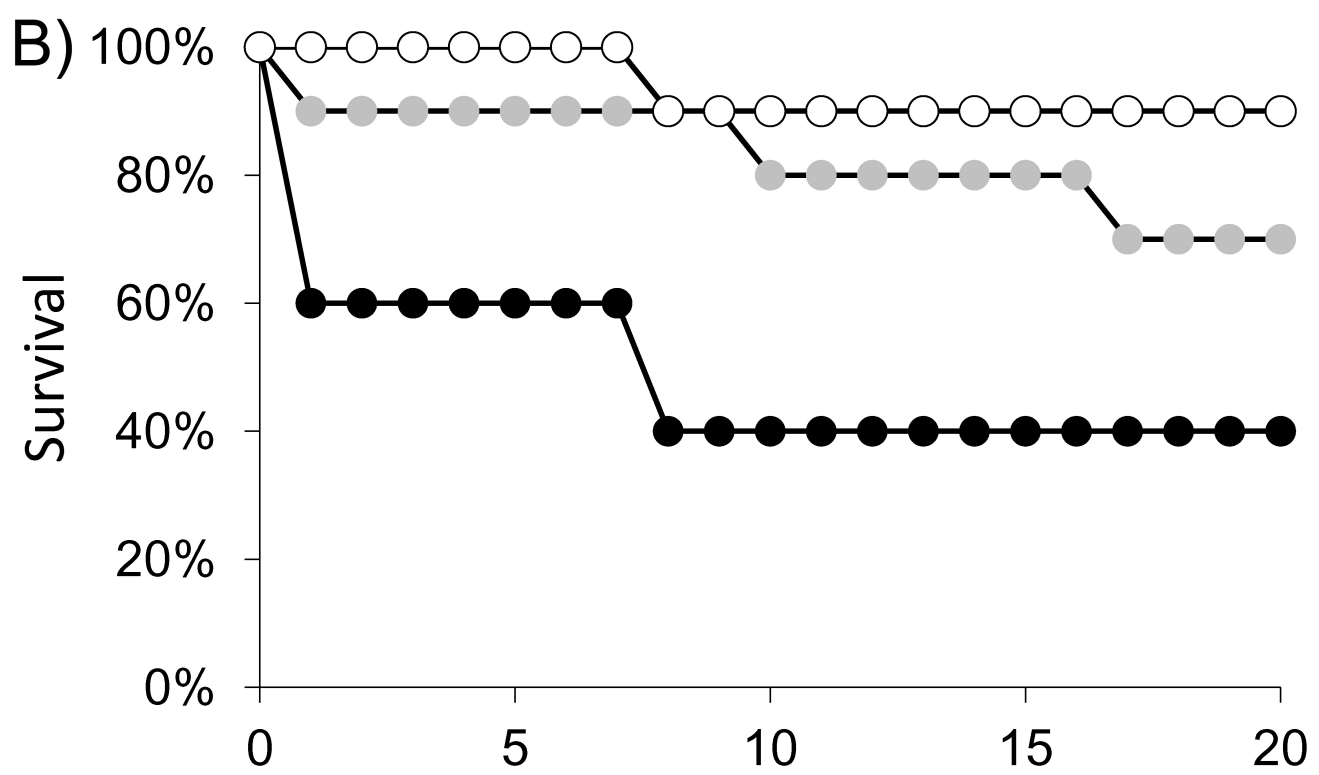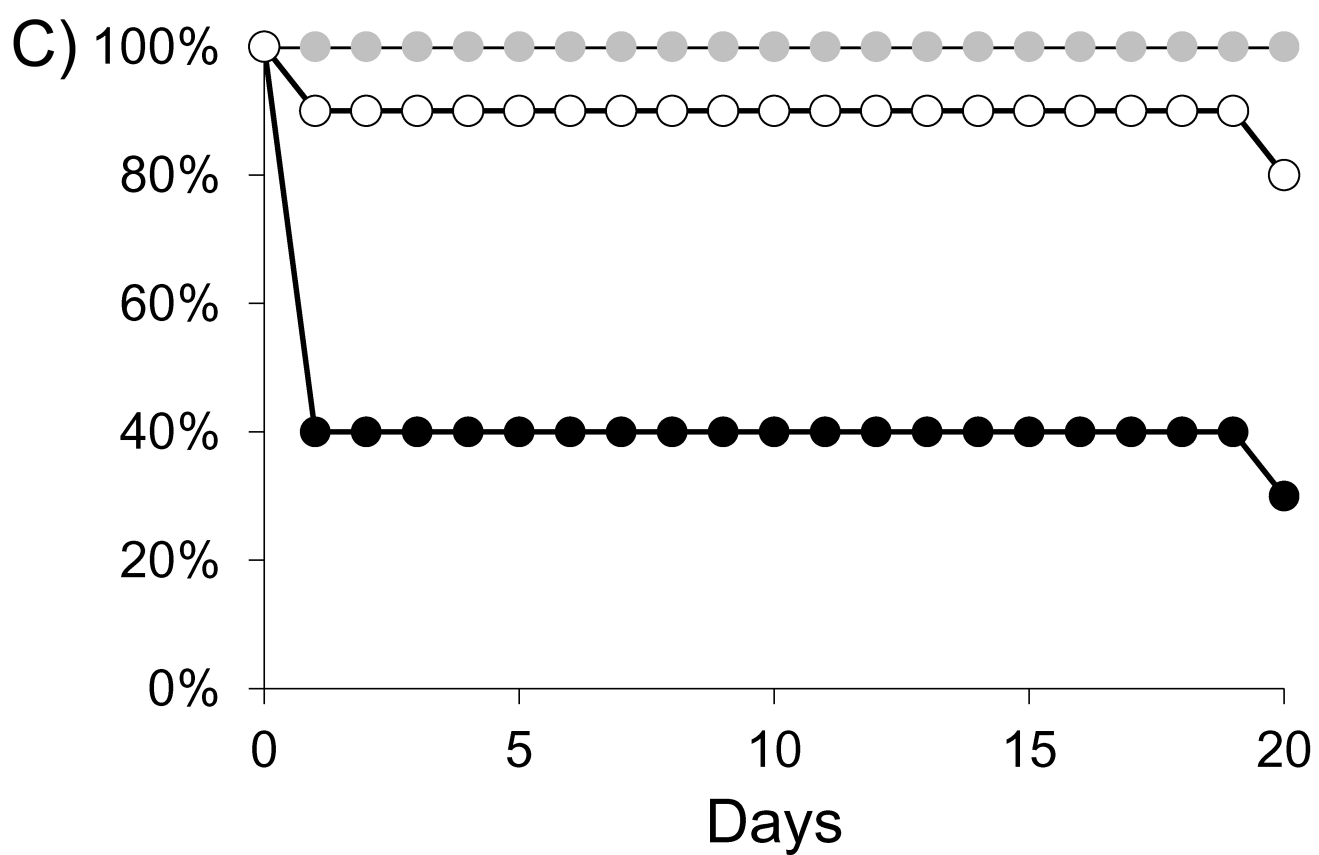

Supplement: S1 Fig — Survival plots of American toads (Anaxyrus [= Bufo] americanus) exposed to (a) a control treatment without Batrachochytrium dendrobatidis (Bd), (b) a low dose of Bd, and (c) a high dose of Bd. Open circles represent toads exposed to the ethanol treatment (n = 10 per treatment), grey circles represent toads exposed to the low CORT treatment (n = 10 per treatment); black circles represent toads exposed to the high CORT treatment (n = 10 per treatment). Exposure to Bd and CORT independently reduced survival, but the two factors did not interact to affect survival. (PDF) [file pone.0163736.s003.pdf]

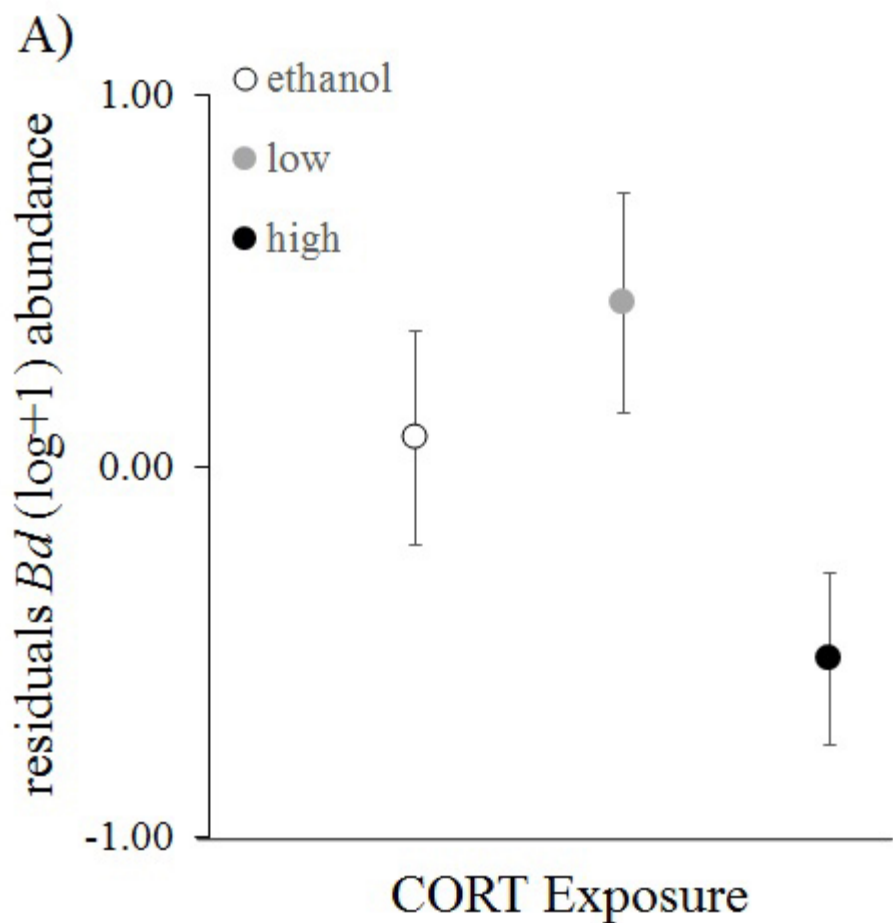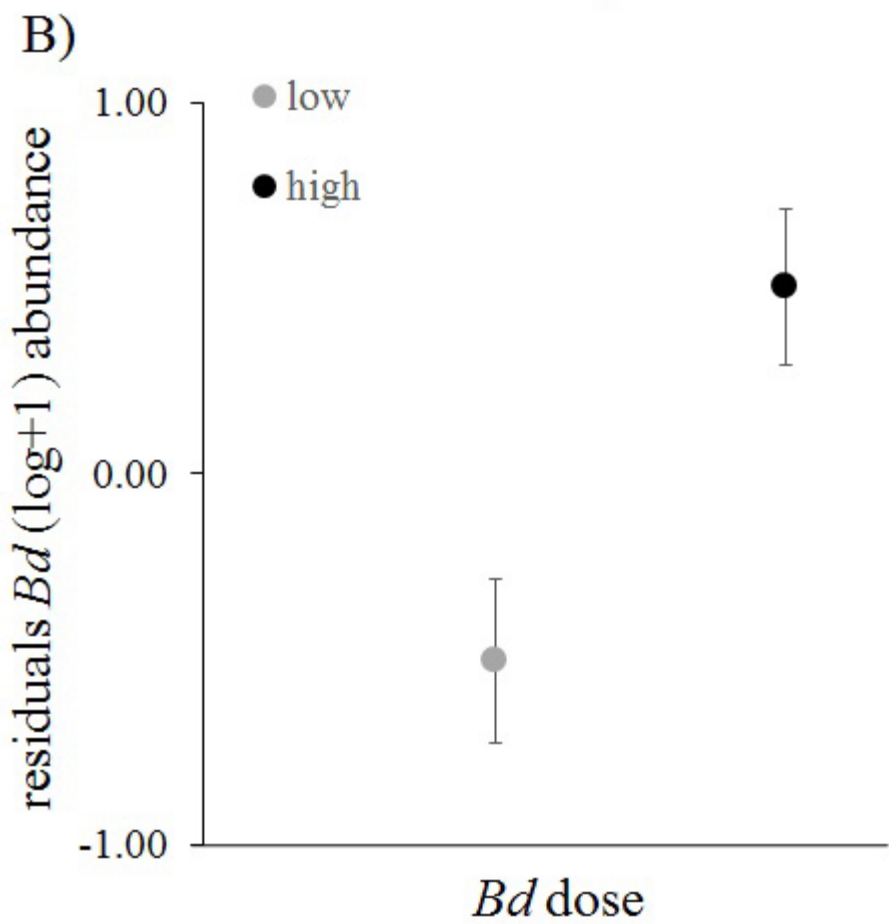

Supplement: S2 Fig — Main effects of (a) CORT exposure and (b) Batrachochytrium dendrobatidis (Bd) dose on American toad (Anaxyrus [= Bufo] americanus) resistance, as measured by Bd abundance. Values represent the average log-transformed Bd abundance for each treatment. Error bars indicate 1 S.E. (PDF) [file pone.0163736.s004.pdf]

% Mass Change

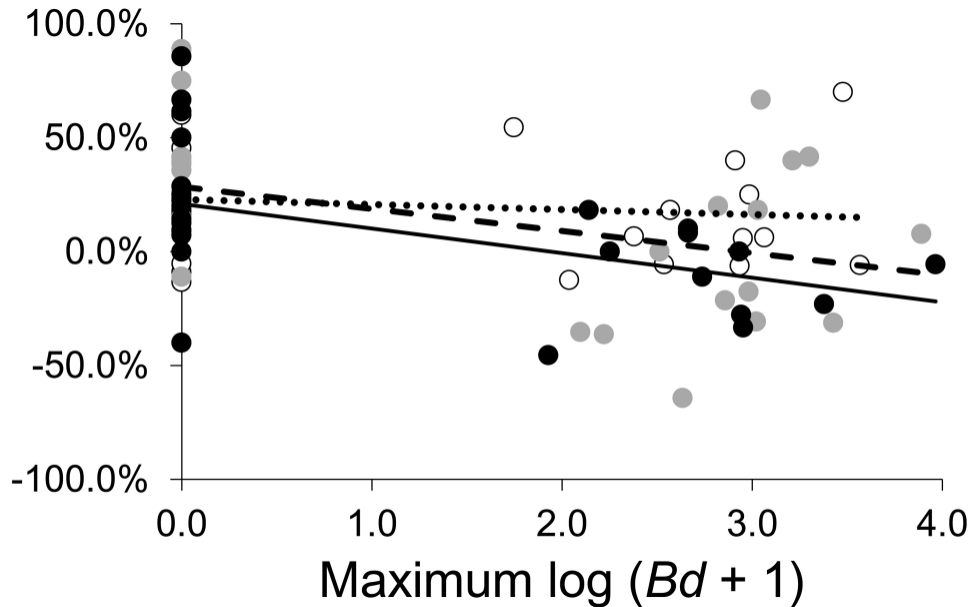

Supplement: S3 Fig — The effect of CORT exposure on American toads (Anaxyrus [= Bufo] americanus) tolerance to Batrachochytrium dendrobatidis (Bd), as measured by the relationship between maximum Bd infection intensity and percentage of mass change. (PDF) [file pone.0163736.s005.pdf]
