# Supplementary material for: Exposure to Corticosterone Affects Host Resistance, but Not Tolerance, to an Emerging Fungal Pathogen
Source: PLoS One. 2016 Sep 30;11(9):e0163736. doi: 10.1371/journal.pone.0163736 (PMC5045185; doi:10.1371/journal.pone.0163736)
Supplement: S1 Table — (PDF) [file pone.0163736.s006.pdf]

S1 Table

Predictor	*F* value	d.f.	*P* value
CORT exposure	7.513	1	0.008
Bd dose	9.990	1	0.002
Mass	3.908	1	0.053
Days Alive	12.203	1	< 0.001
CORT x *Bd*	0.027	1	0.897
Error		54	
